# Transmission of Hepatitis C Virus during Computed Tomography Scanning with Contrast

**DOI:** 10.3201/eid1402.060763

**Published:** 2008-02

**Authors:** Helena Pañella, Cristina Rius, Joan A. Caylà, the Barcelona Hepatitis C Nosocomial Research Working Group

**Affiliations:** * Agència de Salut Pública de Barcelona, Barcelona, Spain

**Keywords:** Hepatitis C virus, computed tomography scanning with contrast, transmission, dispatch

## Abstract

Six cases of acute hepatitis C related to computed tomography scanning with contrast were identified in 3 hospitals. A patient with chronic hepatitis C had been subjected to the same procedure immediately before each patient who developed acute infection. Viral molecular analysis showed identity between isolates from cases with acute and chronic hepatitis C.

The most common mechanism involved in transmission of hepatitis C virus (HCV) is exposure to contaminated blood; sharing of syringes between drug users is currently the most frequent factor. However, in 40% of cases, the mechanism cannot be identified, and nosocomial transmission has an increasingly important role (*1*).

In recent years, transmission of HCV from an infected patient to a susceptible person during various healthcare-related procedures has been reported (*2**,**3*). The risk of transmission depends on the mechanism responsible, as well as on the prevalence of infected persons. Thus, in addition to use of shared vials for administration of heparin (*4*), physiological saline solution (*5**–**7*) or anesthetics (*8**,**9*), procedures carried out with contaminated equipment (*10**,**11*) or inappropriate practices of health personnel (*12*) have been proposed as mechanisms of nosocomial transmission. Molecular analysis of HCV has permitted comparison of different viral sequences to confirm transmission.

Hepatitis C infection is a mandatory reportable disease in Spain and is reported as a suspected infection as soon as it is clinically diagnosed. In Barcelona, after initial reporting of a case of hepatitis C, an epidemiologic survey is conducted by trained personnel from the epidemiologic service to identify possible sources of infection (sexual or household contact with an HCV case, use of multidose medications, transfusions, surgical interventions or other invasive procedures during their hospital stay) and to implement appropriate control measures. The aim of the present study was to describe several cases of nosocomial HCV transmission affecting patients in which the identified possible source of transmission was a computed tomography (CT) scan with contrast in different hospitals in Barcelona in 2004.

## The Study

From August through November 2004, 6 cases of acute hepatitis C associated with a CT scan with contrast were diagnosed in 2 public hospitals and 1 private diagnostic center. Only 1 was detected by active case finding. Three cases were women and 3 were men (ages 29, 47, 55, 57, 58 and 61 years, respectively). All but 1 had symptoms compatible with acute hepatitis with dates of onset from July 8 to September 18, 2004. All had increased serum transaminase levels, positive serologic results for hepatitis C, and HCV RNA detected by reverse transcription–PCR. All cases were investigated and other sources of transmission were ruled out; all participants were outpatients with appointments and did not undergo any specific tests before or after the scan. None shared any equipment or locations with carriers, none received multidose injectable medications or contaminated saline flushes, and none shared other exposures during their CT procedure. All patients had recently undergone a CT scan with contrast: 3 patients on June 11, and 1 each on June 25, June 29, and August 9. An epidemiologic study was conducted that defined a hepatitis C case as a person who satisfied the following 3 criteria: 1) diagnosis of acute hepatitis C according to the case definition of the Catalan Surveillance System (*13*); 2) having undergone a CT scan with contrast in the 6 months before diagnosis; and 3) the case was detected from August through November 2004. All persons tested with the same multidose contrast equipment; a reported case were screened to detect possible HCV carriers.

 Transmission was demonstrated by similarity of HCV sequences isolated from acute hepatitis cases with those sequences isolated from carriers with chronic hepatitis C by using phylogenetic analysis. Briefly, glycoprotein E2 coding sequence (nt 1301–1808 encompassing hypervariable region 1) was amplified by nested PCR as previously described (*3**,**4*). This analysis was performed in 2 laboratories: Hospital Clinic and Hospital Vall d’Hebron.

**Figure 1:**
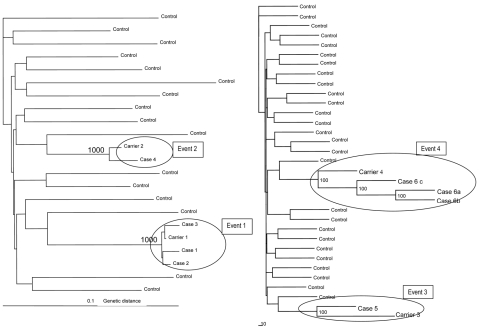
Phylogenetic tree of the partial glycoprotein E2 sequences of hepatitis C virus from patients investigated in 2 public hospitals and 1 private diagnostic center and control samples retrieved from Hospital Clinic and Hospital Vall d’Hebron in Barcelona, Spain. GenBank accession nos.: DQ682391, DQ682392, DQ682393, DQ682394, DQ682376, DQ682377, EU380670, EU380671, EU380672, EU380673, EU380674, EU380675. Branch lengths are drawn to scale. Only bootstrap values >70% are shown.

Four independent carrier-case events were identified, as shown in Table 1. Three cases on the same day were related to the same carrier, 2 cases were identified immediately after the carrier, and 1 case (asymptomatic) was not consecutive (only 1 person between cases 2 and 3), resulting in a secondary attack rate of 60.0% (95% confidence interval [CI) 17.04–92.74) (Table 2) In the other 3 events, the case was scanned immediately after the carrier; global secondary attack rate was 26.08% (95% CI 11.08–48.68). The first event was probably caused by a carrier viral load greater than that of the other events. All cases involved the immediately prior scan of an HCV carrier and they clustered in the same node, on the basis of 1,000 resamplings, with a value ≈100%; some part of the contrast injection equipment had been used for all cases (Figure 1). All transmission events resulted in well-defined, highly supported monophyletic groups when samples involved in the outbreak were analyzed (>95%). Conversely, sequences from unrelated samples did not show bootstrap support (<70%) (*3*,*14*). Epidemiologically unrelated strains from the same area were included in the analysis.

**Table 1:** Characteristics of 6 acute hepatitis C infections attributable to computed tomography (CT) scan with contrast*

Characteristic	Event 1			Event 2	Event 3	Event 4
Hospital/center	A	A	A	A	B	C
Carrier no.	1	1	1	2	3	4
CT scan date/time	2004 Jun 11, 3:00 pm	2004 Jun 11, 3:00 pm	2004 Jun 11, 3:00 pm	2004 Jun 25, 9:05 am	2004 Jun 29, 11:00 am	2004 Aug 9, 5:44 pm
Case-patient no.	1	2	3	4	5	6
Sex	F	M	F	F	M	M
Age, y	47	58	57	55	29	61
CT scan date/time	2004 Jun 11, 3:50 pm	2004 Jun 11, 4:16 pm	2004 Jun 11, 5:17 pm	2004 Jun 25, 9:37 am	2004 Jun 29, 11:29 am	2004 Aug 9, 6:43 pm
Symptom onset	2004 Aug 7	2004 Aug 8	Asymptomatic	2004 Aug 4	2004 Aug 12	2004 Aug 18
Diagnosis	2004 Aug 9	2004 Aug 12	2004 Oct 15	2004 Aug 14	2004 Aug 18	2004 Aug 28
Diagnostic test	Seroconversion, anti-HCV, RNA HCV	Seroconversion, anti-HCV, RNA HCV	Seroconversion, anti-HCV, RNA HCV	Anti-HCV, RNA HCV	Seroconversion, anti-HCV, RNA HCV	Seroconversion, anti-HCV, RNA HCV
Date reported	2004 Aug 20	2004 Aug 20	NA	2004 Aug 20	2004 Nov 22	2004 Nov 5
Genotype	1b, phylogenetic identity with carrier 1	1b, phylogenetic identity with carrier 1	1b, phylogenetic identity with carrier 1	1b, phylogenetic identity with carrier 2	1b, phylogenetic identity with carrier 3	1b, phylogenetic identity with carrier 4

*The 2 hospitals and 1 diagnostic center from which case-patients were identified are designated as A, B, and C. Anti-HCV, antibody to hepatitis C virus; NA, not applicable.

**Table 2:** Transmission of hepatitis C during computed tomography (CT) scan with contrast*

Characteristic	2004			
	Jun 11	Jun 25	Jun 29	Aug 9
No. patients scanned after carrier, sharing same equipment	5	11	5	2
No. cases of transmission	3	1	1	1
Time between carrier scan and case scan	2 h 17 min	32 min	29 min	59 min
Hospital	A	A	B	C
Secondary attack rate, % (95% CI)	60 (17.04–92.74)	9.09 (0.47–42.88)	20 (1.05–70.12)	50 (2.66–97.33)
Syringe replacement	Every 8 h	Every 8 h	<8 h	>24 h
Disconnection procedure with risk of transmission	Present	Present	Present	Present

*CI, confidence interval.

In the hospital where 2 episodes of transmission occurred on different days, a retrospective study was carried out to eliminate other cases of transmission of HCV among the 1,486 patients subjected to a CT scan with contrast after a patient with known HCV infection from May 15 through August 15, 2004. This period included the exposure period of both transmissions. This analysis yielded 71 infectious HCV carriers who had a scan in which equipment was shared with noninfected patients. Of the 71 pairings of carriers-susceptible patients, 57 susceptible persons were tested for antibodies to HCV, 10 had died, and 4 could not be located. No new cases were identified, resulting in a frequency of HCV transmission of 3.5 (95% CI 0.61–13.16) per 100 carriers for whom equipment was shared during the 3-month period. The consecutively scanned patient was HCV negative.

**Figure 2:**
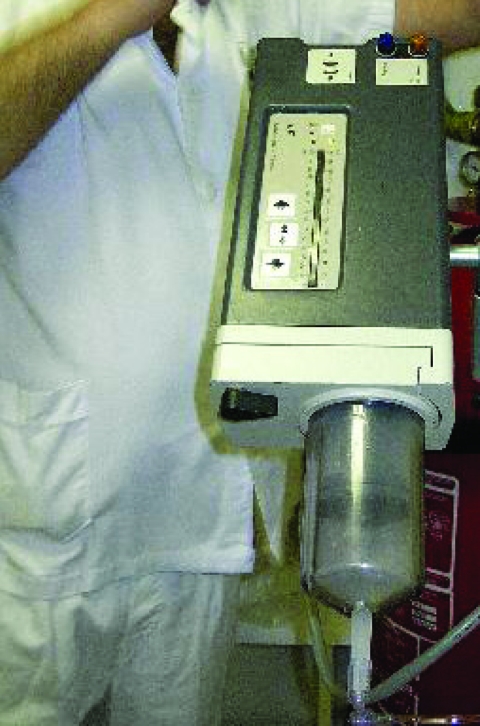
Equipment for contrast administration in computed tomography scan.

Characteristics of the equipment used for contrast injection in all settings were investigated, along with the procedure used, to identify critical points for virus transmission. The equipment (Figure 2) included a contrast injector with automatic load from a 500-mL bottle that was shared by >4 persons. The contrast solution arrived in a prefilled bottle (manufactured by different pharmaceutical companies) that is loaded through a connection tube into the injector. We investigated this procedure and did not detect any risk of blood contamination. Replacement of the injector varied among hospitals, and use time ranged from 8 hours to several days. All equipment was connected to the patient by the intravenous route through an extension tube fitted with a nonreturn valve. This extension tube was the only part of the equipment that was changed for each patient. One maneuver involving risk of blood contamination of the extension tube was identified in all hospitals. Contamination could have occurred through the hands of health personnel manipulating the extension tube by disconnecting the tube from the patient first and then from the equipment without changing gloves between these manipulations. Other factors that could have contributed to transmission were lack of written instructions to guarantee proper practice; training of new technicians by experienced technicians, which may perpetuate errors; and work overload, with a maximum time of <9 min available for each CT scan (from the time the patient enters until he or she leaves), which could lead to a lower level of standard precautions.

Two sets of control measures were used. The first set, which emphasized appropriate use of health products and stressed the importance of following manufacturer’s instructions (a contrast injection syringe for each patient), was obtained from the Agencia Española del Medicamento. The second set included a written protocol for performing a CT scan with contrast and procedures to avoid transmission of infectious diseases. A follow up of infected patients was ensured, as well as preventive treatment. This will be reported by the responsible team in the future.

## Conclusions

Our study had several limitations that should be taken into account. First, our study was a case series and we only described cases and their potential sources of infection. Second, although we carried out active case finding, we must assume that other related cases were not detected. Detection of this problem in 3 hospitals in the same city within a short period suggests that CT scan with contrast could be responsible for an unknown number of preventable HCV infections in industrialized countries if infection control procedures are not strictly applied.

## References

[1] Curry MP, Chopra S. Acute Viral Hepatitis. In: Mandell GL, Bennet JE, Dolin R, editors. Principles and practice of infectious diseases. Philadelphia: Elsevier Churchill Livingstone; 2005. p.1426–41.

[2] Silini E, Locasciulli A, Santoleri L, Gargantini L, Pinzello G, Montillo M, et al.[REMOVED HYPERLINK FIELD] Hepatitis C virus infection in a hematology ward: evidence for nosocomial transmission and impact on hematologic disease outcome. Haematologica. 2002;87:1200–8. 12414351

[3] Nosocomial transmission of HCV in the liver unit of a tertiary care center. Hepatology. 2005;41:115–22.Forns X, Martinez-Bauer E, Feliu A, Garcia-Retortillo M, Martin M, Gay E, et al. 10.1002/hep.20515 15619236

[4] Bruguera M, Saiz JC, Franco S, Giménez-Barcons M, Sanchez-Tapias JM, Fabregas S, Outbreak of nosocomial hepatitis C virus infection resolved by genetic analysis of HCV RNA. J Clin Microbiol. 2002;40:4363–6. 10.1128/JCM.40.11.4363-4366.2002 12409433 PMC139636

[5] Lagging LM, Aneman C, Nenonen N, Brandberg A, Grip L, Norkrans G, et al. Nosocomial transmission of HCV in a cardiology ward during the window phase of infection: an epidemiological and molecular investigation. Scand J Infect Dis. 2002; 34:580–2. 10.1080/00365540110080926 12238573

[6] Dumpis U, Kovalova Z, Jansons J, Cupane L, Sominskaya I, Michailova M, et al. An outbreak of HBV and HCV infection in a paediatric oncology ward: epidemiological investigations and prevention of further spread. J Med Virol. 2003;69:331–8. 10.1002/jmv.10293 12526042

[7] Krause G, Trepka MJ, Whisenhunt RS, Katz D, Nainan O, Wiersma ST, et al. Nosocomial transmission of hepatitis C virus associated with the use of multidose saline vials. Infect Control Hosp Epidemiol. 2003;24:122–7. 10.1086/502176 12602694

[8] Comstock RD, Mallonee S, Fox JL, Moolenaar RL, Vogt TM, Perz JF, et al. A large nosocomial outbreak of hepatitis C and hepatitis B among patients receiving pain remediation treatments. Infect Control Hosp Epidemiol. 2004;25:576–83. 10.1086/502442 15301030

[9] Patient-to-patient transmission of hepatitis C virus through the use of multidose vials during general anesthesia. Infect Control Hosp Epidemiol. 2005;26:789–92.Germain JM, Carbonne A, Thiers V, Gros H, Chastan S, Bouvet E, et al. 10.1086/502618 16209386

[10] Savey A, Simon F, Izopet J, Lepoutre A, Fabry J, Desenclos JC. A large nosocomial outbreak of hepatitis C virus infections at a hemodialysis center. Infect Control Hosp Epidemiol. 2005;26:752–60. 10.1086/502613 16209381

[11] Desenclos JC, Bourdiol-Razes M, Rolin B, Garandeau P, Ducos J, Brechot C, et al. Hepatitis C in a ward for cystic fibrosis and diabetic patients: possible transmission by spring-loaded finger-stick devices for self-monitoring of capillary blood glucose. Infect Control Hosp Epidemiol. 2001;22:701–7. 10.1086/501849 11842991

[12] Bosch X. Newspaper apportions blame in Spanish hepatitis C scandal. Lancet. 2000;355:818. 10.1016/S0140-6736(05)72446-6 10711938

[13] Generalitat de Catalunya. Departament de Sanitat i Seguretat social. Mandatory notifiable disease case definition [in Spanish]. 10th edition. Barcelona; 2005.

[14] Bracho MA, Gosalbes MJ, Blasco D, Moya A, Gonzalez-Candelas F. Molecular epidemiology of a hepatitis C virus outbreak in a hemodiálisis unit. J Clin Microbiol. 2005;43:2750–5. 10.1128/JCM.43.6.2750-2755.2005 15956393 PMC1151931

